# Switch to aflibercept in the treatment of neovascular age-related macular degeneration: 30-month results

**DOI:** 10.5935/0004-2749.20210036

**Published:** 2021

**Authors:** Elif Ertan, Mustafa Doğan, Onur Polat, Neriman Efe, Müberra Akdoğan, Sibel İnan, Reşat Duman, Rahmi Duman

**Affiliations:** 1 Department of Ophthalmology, Gaziosmanpaşa Training and Research Hospital, İstanbul, Turkey; 2 Department of Ophthalmology, School of Medicine, Afyonkarahisar University of Health Sciences, Afyonkarahisar, Turkey; 3 Department of Ophthalmology, Bursa Dünya Göz Hospital, Bursa Turkey; 4 Department of Ophthalmology, Bursa City Hospital, Bursa, Turkey; 5 Department of Ophthalmology, Liv Hospital, Ankara, Turkey

**Keywords:** Macular degeneration, Ranibizumab/therapeutic use, Angiogenesis inhibitors/therapeutic use, Intravitreal injections, Retina/pathology, Visual acuity, Degeneração macular, Ranibizumab/uso terapêutico, Inibidores de angiogênese /uso terapêutico, Injeções intravítreas, Retina/patologia, Acuidade visual

## Abstract

**Purpose:**

This study was conducted to evaluate visual function and changes in the central macular thickness of patients with unresponsive neovascular age-related macular degeneration who were switched from ranibizumab (Lucentis^®^) to aflibercept (Eylea^®^) treatment at 30 months.

**Methods:**

This retrospective study examined patients with neovascular age-related macular degeneration who were switched to aflibercept after ≥6 previous intravitreal ranibizumab injections at 4- to 8-week intervals. All patients were switched to intravitreal aflibercept (2.0 mg) and analyzed after 3 consecutive injections followed by a prore nata dosing regimen and after 30 months of treatment. Best corrected visual acuity, biomicroscopic examination, intraocular pressure, fundus examination, and central macular thickness were recorded at the start of treatment, before the transition to intravitreal aflibercept treatment, and at 6, 12, 18, 24, and 30 months of intravitreal aflibercept treatment.

**Results:**

A total of 33 eyes met the inclusion criteria. The median age of the patients was 73.57 ± 7.98 years, and 21 (61.8%) patients were males and 12 (35.3%) were females. Before the transition, the patients received a mean of 16.8 ± 8.8 ranibizumab injections (range 6-38).After the transition to intravitreal aflibercept treatment, the mean number of aflibercept injections was 9.09 ± 3.94. No significant differences were observed in best corrected visual acuity after the aflibercept switch in any of the months. The central macular thickness was significantly decreased at 6, 12, 18, and 30 months (p=0.01, p=0.03, p=0.05, p=0.05, p<0.001, respectively).

**Conclusıon:**

Patients with neovascular age-related macular degeneration who were switched to intravitreal aflibercept treatment due to unresponsiveness to intravitreal ranibizumab exhibited a significant anatomic improvement in the retina, and although this state persisted, there was no significant functional gain.

## Abstract (pt)

**Objetivo:**

Avaliar, depois de 30 meses, a função visual e as alterações na espessura macular central de pacientes com degeneração macular relacionada à idade sem resposta terapêutica ao ranibizumabe (Lucentis^®^) que mudaram seu tratamento para o aflibercepte (Eylea^®^).

**Métodos:**

Realizou-se um estudo retrospectivo de pacientes com degeneração macular neovascular relacionada à idade que mudaram o tratamento para o aflibercepte após 6 ou mais injeções intravítreas de ranibizumabe a intervalos de 4-8 semanas. Todos os pacientes mudaram para o aflibercepte intravítreo (2,0 mg) e depois de 3 injeções consecutivas, seguidas de um regime de dosagem *pro re nata*, foram avaliados após 30 meses de tratamento. A melhor acuidade visual corrigida, o exame biomicroscópico, a pressão intraocular, a fundoscopia e a espessura macular central foram registrados no início do tratamento, antes da transição para o tratamento com aflibercepte intravítreo e aos 6, 12, 18, 24 e 30 meses de tratamento com o aflibercepte intravítreo.

**Resultados:**

Satisfizeram aos critérios de inclusão 33 olhos. A mediana da idade dos pacientes foi de 73,57 ± 7,98 anos. Dos pacientes, 21 (61,8%) eram homens e 12 (35,3%) eram mulheres. Antes da transição para o tratamento com o aflibercepte intravítreo, os pacientes receberam em média 16,8 ± 8,8 injeções de ranibizumabe (faixa 6-38).Depois da transição, o número médio de injeções de aflibercepte foi de 9,09 ± 3,94. Não houve diferenças significativas na melhor acuidade visual corrigida depois da mudança para o aflibercepte em qualquer das avaliações. Houve diminuição significativa da espessura macular central aos 6, 12, 18 e 30 meses (respectivamente, p=0,01, p=0,03, p=0,05, p=0,05 e p<0,001).

**Conclusão:**

Pacientes com degeneração macular neovascular relacionada à idade que mudaram seu tratamento para o aflibercepte intravítreo devido à falta de resposta ao ranibizumabe intravítreo, tiveram melhora anatômica significativa da retina; mas embora esse estado tenha persistido, não foi observado nenhum ganho funcional significativo.

## INTRODUCTION

Age-related macular degeneration (AMD) is a chronic degenerative disease and the leading cause of blindness in elderly individuals living in developed countries^(1)^. Intravitreal anti-vascular endothelial growth factor (VEGF) agents have become the gold standard in the treatment of neovascular AMD based on the understanding that VEGF plays a key role in age-related AMD neovascularization^(2)^.

Several multicenter studies have demonstrated the efficacy and safety of intravitreal ranibizumab treatment in n-AMD^(3-5)^. Although monthly ranibizumab or pro re nata (PRN) use was found to preserve visual acuity in 90% of patients, increase in visual acuity was reported in 30% of patients^(3-5)^. Despite the positive results reported in multicenter trials, AMD remains a chronic disease requiring long-term follow-up and recurrent intravitreal injections. Patients treated with ranibizumab may have persistent edema or reactivation of the disease despite repeated intravitreal injections^(6)^.

Aflibercept (Eylea^®^; Regeneron Pharmaceuticals, Tarrytown, NY, USA) is one of the anti-VEGF agents exhibiting a higher affinity to VEGF, a longer half-life, and the capability of inhibiting not only VEGF-A, which is also inhibited by ranibizumab, but also VEGF-B and placental growth factor^(7-10)^. Based on the result of VIEW 1 and 2 studies, aflibercept was approved by the Food and Drug Administration (FDA) for the treatment of n-AMD^(11)^.

Switching the anti-VEGF agent is a commonly used approach in cases with persistent fluid or multiple recurrences during ranibizumab treatment^(12,13)^. Some clinical studies report that refractory patients who were switched to aflibercept from bevacizumab/ranibizumab had better functional and anatomical outcomes^(14,15)^. We had also previously reported the 18-month outcomes of patients with n-AMD resistant to ranibizumab treatment who were switched to aflibercept^(16)^.

In the present study, we evaluated the visual and anatomical outcomes of switching from ranibizumab to aflibercept therapy in patients with n-AMD at 30 months.

## METHODS

In this retrospective study, we reviewed the medical records of all patients with n-AMD treated at Afyon Kocatepe University Hospital, Turkey, a tertiary care center, between January 2014 and June 2018. Our study was approved by the local Ethical Committee and conducted in accordance with the Declaration of Helsinki. Written informed consent was obtained from all study participants. Patients with a known diagnosis of n-AMD who were resistant to intravitreal ranibizumab injection with PRN regimen and switched to aflibercept treatment were included in this study.

Inclusion criteria for our study were as follows: the presence of n-AMD previously treated with intravitreal ranibizumab that was switched to intravitreal aflibercept, persistent intraretinal or subretinal fluid, a minimum of 6 ranibizumab injections with PRN regimen before the transition, the last injection of ranibizumab being administered within 28-35 days of switching to aflibercept, and at least 30 months of follow-up after the switch to aflibercept. Exclusion criteria were history of vitrectomy, choroidal neovascularization (CNV) lesions secondary to causes other than AMD, the presence of -6.00 D or greater myopia, uncontrolled glaucoma, uveitis, or any other ocular disease that could potentially confound the assessment of safety and/or efficacy of treatment.

A total of 33 patients met the inclusion criteria and were analyzed in this study. The indication for transition to aflibercept was inadequate response to ranibizumab, which was defined as persistent or recurrent subretinal and/or intraretinal fluid. Persistent subfoveal fluid was defined as the presence of intraretinal/subretinal fluid at the fovea or subfoveal subretinal pigment epithelium fluid with adjoining subretinal/intraretinal fluid, and recurrent subretinal and/or intraretinal fluid was defined as exudation suppressed with treatment, but upon suspension, exudation recurred. During the monthly follow-up period, patients underwent complete ophthalmologic examinations, including best corrected visual acuity (BCVA) using Early Treatment Diabetic Retinopathy Study (ETDRS) charts, intraocular pressure (IOP) measurement, fundus examination, and spectral domain optical coherence tomography (SD-OCT, Heidelberg Spectralis, Heidelberg Engineering, Heidelberg, Germany) scanning. The same ophthalmologist (EE) performed the first visit’s SD-OCT and fundus fluorescein angiography (FFA) evaluations. The CNV lesions were graded as occult, predominantly classic, minimally classic, polypoidal choroidal vasculopathy, or retinal angiomatous proliferation based on FFA findings.

After switching from ranibizumab, all patients received a loading dose of 3 monthly aflibercept injections (2 mg/0.05 ml) and were followed up monthly. Retreatment with a single aflibercept injection was performed on the basis of any of the following findings: visual acuity loss of at least 5 letters with SD-OCT evidence of fluid in the macula, persistent or recurrent intraretinal or subretinal fluid in SD-OCT, or new subretinal hemorrhage from CNV.

Demographic characteristics of the patients were recorded. The primary outcomes of the study were variations in BCVA and central macular thickness (CMT) after switching to aflibercept at 0, 6, 12, 18, and 30 months and the frequency of aflibercept injections.

The obtained data were analyzed using the statistical package program (SPSS, version 18.0, SPSS, Chicago, USA). The distribution of variables was evaluated using the Kolmogorov-Smirnov test. Paired *t*-test was used to compare the measurements obtained before and during injections. The evaluations were made at the 95% confidence interval, and p<0.05 was considered to be statistically significant.

## RESULTS

A total of 33 eyes met the inclusion criteria. The median age of the patients was 73.57 ± 7.98 years. There were 21 (61.8%) male patients 12 (35.3%) female patients. The mean treatment time before the switch was 30.18 ± 16.78 months. The mean number of ranibizumab injections before the switch was 16.8 ± 8.8 (range 6-38).The mean number of aflibercept injections after the switch was 9.09 ± 3.9 (range 3-16). Table 1 shows the baseline characteristics of the patients who were switched to aflibercept.

**Table 1:** Patients characteristics

Total patients/eyes, n/n	33/33
Male, n (%)	21 (61.8%)
Age, years (range)	73.57 (58-86)
Average prior follow-up period, months (range)	30.18 (8-56)
Average prior injections ± SD (range)	16.8 ± 8.8 (6-38)
Average aflibercept injections ± SD (range)	9.09 ± 3.9 (3-16)
Angiographic classification, n (%) Occult with no classic	17 (51.5)
Predominantly classic	10 (30.3)
Minimally classic	6 (18.2)

The CMT and BCVA measurements from baseline to 30 months in all patients are presented in table 2. A significant decrease in CMT was observed at 6, 12, 18, and 30 months (p=0.01, p=0.03, p=0.05, p=0.05, p<0.001, respectively). BCVA measurements showed no statistically significant difference during IVA treatment (p=0.07, p=0.36, p=0.34, p=0.81, p=0.58, respectively). The last SD-OCT imaging obtained at the 30^th^ month showed no subretinal or intraretinal fluid in 23 patients (69.7%). During the study period, neither systemic complications such as cardiovascular or cerebrovascular events nor ocular complications such as endophthalmitis, vitreous hemorrhage, retinal detachment, and sustained IOP increase were observed.

**Table 2:** CMT and BCVA values of the patients

	CMT mean ± SD (IQR)	p value	BCVA mean ± SD (IQR)	p value
Baseline	351.4 ± 126.1 (243.0-438.5)	-	1.08 ± 0.53 (0.60-1.60)	-
6 months	284.8 ± 112.6 (208.5-345.0)	0.01	0.91 ± 0.46 (0.57-1.30)	0.07
12 months	296.7 ± 89.2 (232.0-350.5)	0.03	1.14 ± 0.59 (0.52-1.80)	0.36
18 months	282.6 ± 81.6 (210.7-329.2)	0.05	0.94 ± 0.55 (0.40-1.58)	0.34
24 months	292.3 ± 109.9 (222.5-341.5)	0.05	1.07 ± 0.49 (0.62-1.51)	0.81
30 months	269.7 ± 97.1 (207.7-330.0)	<0.001	1.15 ± 0.57 (0.60-1.51)	0.58

Baseline= Before switch; CMT= central macula thickness; BCVA= Best corrected visual acuity; IQR= Interquartile range; p= paired t test.

## DISCUSSION

This study demonstrated that patients with n-AMD who were unresponsive to ranibizumab treatment and had persistent fluid or recurrence and were switched to intravitreal aflibercept treatment had anatomical improvement of the macula, which continued throughout the 30-month follow-up period. However, the improvement in visual acuity was not as successful as the anatomical improvement.

Several reasons may be proposed to explain the ana tomical improvement after the transition to aflibercept treatment. The first concern could be the pharmaco dynamics of the drug. Tachyphylaxis, defined as a di mi nished therapeutic response to a pharmacologic agent following repeated administrations over time, to ranibizumab may have developed; therefore, there may be a lack of response to the drug despite the high drug concentration attained with frequent repeated injections in short time intervals^(17)^. However, the drug may regain its efficacy when treatment is suspended for a short period of time. Another cause may be the development of tolerance to ranibizumab, wherein long-term use may lead to a significant decrease in the extent and duration of drug efficacy. Unlike tachyphylaxis, drug efficacy cannot be regained by suspending treatment. One study demonstrated that 81% of patients with tachyphylaxis who were switched from bevacizumab to ranibizumab showed a positive response^(18)^. Furthermore, antibodies against the injected agent may have developed in patients who developed a local or systemic immune response after intravitreal injections. Studies also suggest that chronic VEGF blockage leads to excessive macrophage-mediated VEGF production in choroidal neovascular tissue^(19,20)^.

Pharmacological structure of the molecule may also be one of the causes for the anatomical improvement after the switch to aflibercept. Although ranibizumab is a human IgG1 isotype, the integration of VEGF receptors 1 and 2 and IgG crystalline fragments causes the monoclonal antibody aflibercept to exhibit 100 times higher VEGF-A-binding affinity^(21,22)^. Aflibercept also inhibits other factors such as VEGF-B and placental growth factor that affect neovascularization^(7-9)^. It has also been demonstrated that aflibercept has a half-life of 7.13 days in vitreous fluid and an intraocular duration of action of 48-80 days, which is longer than those of ranibizumab (vitreous half-life 4.75 days, intraocular duration of action 28-67 days)^(10)^.

Aflibercept has also been reported to have longer duration and higher binding affinity, and therefore lower dose and fewer injections are required^(24)^. Similarly, in our study, the average number of injections was 16 before the switch to aflibercept and 9 after the switch, despite the similar mean follow-up period^(21-23)^.

Although some studies have reported visual improvement after the switch, several studies report the opposite^(24-27)^. In our study, although the improvement in retinal thickness was apparent, this was not the case in terms of visual function. This inconsistency between the anatomical and visual outcomes might be explained by the chronic structural changes of the macula and the loss of foveal photoreceptors. The literature shows that intravitreal injections used as initial treatment for n-AMD lead to visual improvement that is later followed by a plateau stage, in which chronic degeneration of photoreceptors does not allow visual improvement to accompany monthly injections^(28)^. On the other hand, chronic intrafoveal fluid has negative effects on the cell morphology and function of photoreceptors^(26,28)^. Long-term use of anti-VEGF may also increase the risk of geographical atrophy^(28)^. In our study, 23 (69.7%) patients who were followed up for 30 months had dry macula in their final examination, and 3 (13.1%) of these patients had developed geographic atrophy. All these factors may have contributed to the disappointing level of visual recovery, although a larger series of studies is necessary to support our results.

Possible limitations of our study were the small sample size of patients and the PRN protocol of aflibercept administration. However, we believe that the results obtained from the prospective planning of the study protocol and the long-term follow-up of patients will contribute to the literature.

In conclusion, switching to aflibercept treatment resulted in anatomical recovery in the initial months of therapy that continued for a long period of time in patients with n-AMD who had recurrence or were unresponsive to intravitreal ranibizumab, but there was no functional improvement.

## References

[1] Lim LS, Mitchell P, Seddon JM, Holz FG, Wong TY. (2012). Age-related macular degeneration. Lancet.

[2] Abraham P, Yue H, Wilson L. (2010). Randomized, double-masked, sham-controlled trial of ranibizumab
for neovascular age-related macular degeneration: PIER Study year
2. Am J Ophthalmol.

[3] Brown DM, Kaiser PK, Michels M, Soubrane G, Jeier JS, Kim RY, Sy JP, Schneider S, ANCHOR Study Group (2006). Ranibizumab versus verteporfin for neovascular age-related
macular degeneration. N Engl J Med.

[4] Rosenfeld PJ, Brown DM, Heier JS, Boyer DS, Kaiser PK, Chung CY, Kim RY, MARINA Study Group (2006). Ranibizumab for neovascular age-related macular
degeneration. N Engl J Med.

[5] Lalwani GA, Rosenfeld PJ, Fung AE, Dubovy SR, Michels S, Feuer W (2009). A variable-dosing regimen with intravitreal ranibizumab for
neovascular age-related macular degeneration: year 2 of the PrONTO
Study. Am J Ophthalmol.

[6] Krebs I, Glittenberg C, Ansari-Shahrezaei S, Hagen S, Steiner I, Binder S. (2013). Non-responders to treatment with antagonists of vascular
endothelial growth factor in age-related macular
degeneration. Br J Ophthalmol.

[7] Stewart MW, Rosenfeld PJ. (2008). Predicted biological activity of intravitreal VEGF
Trap. Br J Ophthalmol.

[8] Stewart MW, Rosenfeld PJ, Penha FM, Wang F, Yehoshua Z, Bueno-Lopez E (2012). Pharmacokinetic rationale for dosing every 2 weeks versus 4 weeks
with intravitreal ranibizumab, bevacizumab, and aflibercept (vascular
endothelial growth factor Trap-eye). Retina.

[9] Papadopoulos N, Martin J, Ruan Q, Rafique A, Rosconi MP, Shi E (2012). Binding and neutralization of vascular endothelial growth factor
(VEGF) and related ligands by VEGF Trap, ranibizumab and
bevacizumab. Angiogenesis.

[10] Browning DJ, Kaiser PK, Rosenfeld PJ, Stewart MW. (2012). Aflibercept for age-related macular degeneration: a game-changer
or quiet addition?. Am J Ophthalmol.

[11] Heier JS, Brown DM, Chong V, Korobelnik JF, Kaiser PK, Dong QN, Kirchohof B, Ho A, Yuichiro O, Yancopulos GD, Stahl N, Vitti R, Berliner AJ, Soo Y, Anderesi M, Groetzbach G, Alyson VI, Sommerauer B, Sandbrink R, Simader C, Schmidt-Erfurth U, VIEW 1 and VIEW 2 Study Groups. (2012). Intravitreal aflibercept (VEGF Trap-Eye) in wet age-related
macular degeneration. Ophthalmology.

[12] Bakall B, Folk JC, Boldt HC, Sohhn EH, Stone EM, Russell SR (2013). Aflibercept therapy for exudative age-related macular
degeneration resistant to bevacizumab and ranibizumab. Am J Ophthalmol.

[13] Cho H, Shah CP, Weber M, Heier JS. (2013). Aflibercept for exudative AMD with persistent fluid on
ranibizumab and/or bevacizumab. Br J Ophthalmol.

[14] Wykoff CC, Brown DM, Maldonado ME, Croft DE. (2014). Aflibercept treatment for patients with exudative age-related
macular degeneration who were incomplete responders to multiple ranibizumab
injections (TURF trial). Br J Ophthalmol.

[15] Chang AA, Li H, Broadhead GK, Hong T, Schlub TE, Wijeyakumar W (2014). Intravitreal aflibercept for treatment-resistant neovascular
age-related macular degeneration. Ophthalmology.

[16] Ertan E, Dogan M. Neovascular age-related macular degeneration: 18-month outcomes
of aflibercept treatment in patients resistant to
ranibizumab. Eur Res J.

[17] Brown DM, Chen E, Mariani A, MajorJ r. JC, SAVE Study Group (2013). Super-dose Anti-VEGF (SAVE) Trial: 2.0 mg Intravitreal
ranibizumab for recalcitrant neovascular macular degeneration-primary end
point. Ophthalmology.

[18] Gasperini JL, Fawzi AA, Khondkaryan A, Lam L, Chong LP, Elliot D (2012). Bevacizumab and ranibizumab tachyphylaxis in the treatment of
choroidal neovascularization. Br J Ophthalmol.

[19] Grossniklaus HE, Ling JX, Wallace TM, Dithmar S, Lawson DH, Cohen C (2002). Macrophage and retinal pigment epithelium expression of
angiogenic cytokines in choroidal neovascularization. Mol Vis.

[20] Espinosa-Heidmann DG, Suner IJ, Hernandez EP, Monroy D, Csaky KG, Cousins SW. (2003). Macrophage depletion diminishes lesion size and severity in
experimental choroidal neovascularization. Invest Ophthalmol Vis Sci.

[21] Fassnacht-Riederle H, Becker M, Graf N, Michels S. (2014). Effect of aflibercept in insufficient responders to prior
anti-VEGF therapy in neovascular AMD. Graefes Arch Clin Exp Ophthalmol.

[22] Avery RL, Castellarin AA, Steinle NC, Dhoot DS, Pieramici DJ, See R (2014). Systemic pharmacokinetics following intravitreal injections of
ranibizumab, bevacizumab or aflibercept in patients with neovascular
AMD. Br J Ophthalmol.

[23] Heier JS, Boyer D, Nguyen QD, Marcus D, Roth DB, Yancopous G, Stahl N, Ingerman A, Vitti R, Bernier AJ, Yang K, Brown DM, CLEAR-IT 2 Investigators. (2011). The 1-year results of CLEAR-IT 2, a phase 2 study of vascular
endothelial Trap-Eye dosed as needed after 12-week fixed
dosing. Ophthalmology.

[24] Kumar N, Marsiglia M, Mrejen S, Fung TC, Slakter J, Sorenson J (2013). Visual and anatomical outcomes of intravitreal aflibercept in
eyes with persistent subfoveal fluid despite previous treatments with
ranibizumab in patients with neovascular age-related macular
degeneration. Retina.

[25] Arcinue CA, Ma F, Barteselli D, Sharpsten L, Gomez ML, Freeman WR. (2015). One-year outcomes of aflibercept in recurrent or persistent
neovascular age-related macular degeneration. Am J Ophthalmol.

[26] Pinheiro-Costa J, Costa JM, Beato JN, Freitas-da-Costa P, Brandão E, Falção MS (2015). Switch to aflibercept in the treatment of neovascular AMD:
One-year results in clinical practice. Ophthalmologica.

[27] Jørstad ØK, Faber RT, Moe MC. (2017). Two-year functional and anatomical results after converting
treatment resistant eyes with exudative age-related macular degeneration to
aflibercept in accordance with a treat and extend protocol. Acta Ophthalmol.

[28] Martin DF, Maguire MG, Fine SL, Ying GS, Jaffe GJ, Grunwald JE, Toth C, Redford M, Ferris 3rd FL, Comparison of Age-related Macular Degeneration Treatments Trials
(CATT) Research Group (2012). Ranibizumab and bevacizumab for treatment of neovascular
age-related macular degeneration: two-year results. Ophthalmology.

